# The Effects of Different Organic Amendment Strategies on Soil Properties and Microbial Communities in Maize Monocropping

**DOI:** 10.3390/plants15121805

**Published:** 2026-06-11

**Authors:** Ming Fang, Jianan Sun, Xinyue Li, Jiaming Zhang, Chuyi Wang, Shuxuan Qi, Yixin Guan, Qiang Lyu, Gang Yang, Man Ao, Yubo Zhu, Bo Li

**Affiliations:** 1 Jilin Provincial Key Laboratory of Plant Resource Science and Green Production, Jilin Normal University, Siping 136000, China; 2 Northeast Institute of Geography and Agroecology, Chinese Academy of Sciences, Changchun 130102, China; 3 College of Food Science and Engineering, Boda College of Jilin Normal University, Siping 136000, China; 4 College of Land Science and Technology, China Agricultural University, Beijing 100193, China; 5 Jilin Lishu Experimental Station, China Agricultural University, Siping 136500, China

**Keywords:** black soils, organic materials, soil microbial community composition, straw returning

## Abstract

The black soil layer has undergone substantial degradation in Northeast China, and it is crucial to adopt reasonable tillage measures to prevent black soil degradation. Organic amendment strategies provide an effective solution for mitigating nutrient loss in black soil; meanwhile, there is still a lack of systematic investigation into their impact on soil microbial communities. Thus, we carried out a five-year field experiment from 2020 to 2025 in Jilin Province. Four organic amendment strategies were set up: conventional tillage (CT); straw returning (SR); straw returning + inorganic fertilizer (SRI); and straw returning + inorganic fertilizer + organic fertilizer (SRIO). Furthermore, we investigated the effects of organic materials on soil properties and microbial communities during the maize seedling stage. The results showed that SR significantly increased the relative abundance of *Bradyrhizobium*, *Tausonia* and *Coprinopsis*, while SRI led to a 140.3% increase in *Nocardioides*. In SRIO treatment, *Gaiella* and *Fusarium* were significantly enriched by 103.9% and 142.5%, respectively. Moreover, SR treatment significantly decreased the fungal Shannon and Simpson index by 18.8% and 4.2%, respectively. Organic matter, alkali nitrogen, and available potassium were the primary environmental factors shaping both bacterial and fungal community structures. Additionally, the co-occurrence network suggested that straw returning promoted more diverse interactions among soil bacterial and fungal communities. Our study highlights the potential of organic amendment strategies in enhancing black soil nutrients, as well as their important role in maintaining soil microbial function and stability.

## 1. Introduction

The organic-rich composition and unique physical properties make black soil one of the world’s most productive soils. The black soil region in Northeast China plays a critical role in ensuring food security and sustainability [1]. However, due to intensive land use, unsustainable farming practices, and agrochemical applications, the black soil layer has undergone substantial degradation. The average thickness has sharply decreased from 60–80 cm to 20–30 cm since the 1950s, posing a serious threat to agricultural production [2]. Meanwhile, black soil degradation is accompanied by organic matter depletion, severe erosion, acidification, and compaction issues [3]. Therefore, it is crucial to adopt reasonable tillage measures to prevent black soil degradation.

Chemical fertilizers are used primarily to provide essential nutrients to plants, promoting their growth and enhancing crop yields. However, they also lead to serious ecological issues such as soil contamination and biodiversity loss [4]. Simultaneously, nutrient losses through leaching and volatilization have contributed to a decline in nutrient use efficiency [5]. Straw returning is a sustainable management practice that improves soil fertility and productivity [6]. For example, studies have shown that the longer duration of straw returning increased the contents of soil organic matter, available phosphorus, total nitrogen, and alkaline nitrogen [7,8,9]. Consequently, straw returning can mitigate nutrient loss and serve as a comprehensive protection technology for black soil.

Straw returning could lead to changes in the microbial community composition and structure, and soil microorganisms are important for the nutrient cycling, energy flow, and the maintenance of soil health and ecological balance [10,11]. Different tillage practices can regulate microbial diversity and function. For example, *Schizothecium* (fungi) and *Massilia* (bacteria) were enriched in straw returning and manure fertilization treatments, both of which were associated with organic matter decomposition and nitrogen cycling [2]. Furthermore, the relative abundance of genes involved in the solubilization and mineralization of phosphorus were significantly increased by straw returning [12,13]. The increase in microbial diversity might be attributed to the buffering effect of straw returning on soil temperature and moisture fluctuations [14]. Further research showed that microbial community structure was significantly correlated with soil metabolites [15]. In agricultural practices, straw returning is less effective under conditions of severe nutrient depletion and organic matter loss, yet the cost of its implementation is high [16]. Organic amendment strategies, such as combining organic fertilization with straw returning, could effectively retain water and nutrients, thereby supporting beneficial microorganisms [17]. This cultivation mode not only enhanced soil carbon sequestration but also reduced greenhouse gas emissions such as N_2_O by altering microbial community metabolisms [5,18]. However, the response of microbial diversity to organic materials remains insufficiently explored [2,18]. It is necessary to investigate the response of microbial community to different organic amendments, which will provide a basis for optimizing the combination of straw and fertilizer resources to mitigate black soil degradation.

Thus, a five-year field experiment was conducted in the Northeast China, where the soil is classified as black soil. Four treatments were set up: conventional tillage (CT), straw returning (SR), straw returning combined with inorganic fertilizer (SRI), and straw returning combined with inorganic and organic fertilizer (SRIO). This study systematically evaluated the effects of different organic amendment strategies on soil chemical properties and microbial communities. Our research will provide insights into strategies for the protection and sustainable utilization of black soil resources.

## 2. Results

### 2.1. Effects of Organic Amendment Strategies on Soil Properties

**Figure 1:**
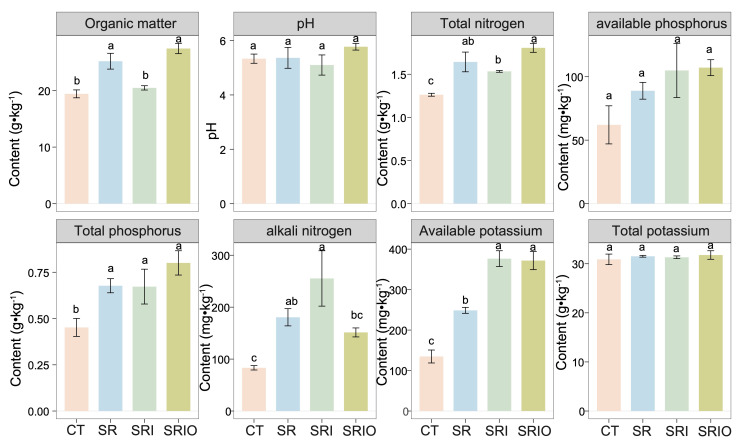
Effects of organic amendment strategies on soil chemical properties. Values are represented as means ± standard errors (n = 3). Different letters indicated significant differences among treatments (one-way ANOVA, Duncan’s test, *p* < 0.05). CT: conventional tillage; SR: straw returning; SRI: straw returning + inorganic fertilizer; SRIO: straw returning + inorganic fertilizer + organic fertilizer.

Straw returning (SR) significantly enhanced the content of soil organic matter, total nitrogen, alkali nitrogen, total phosphorus and available potassium. Generally, compared with SR treatment, a significant decrease in total nitrogen and alkali nitrogen contents were observed under the addition of inorganic fertilize treatment (SRI). Moreover, the organic materials had no significant effect on soil pH, available phosphorus and total potassium (Figure 1).

### 2.2. Effects of Organic Amendment Strategies on Soil Microbial Community Composition

**Figure 2:**
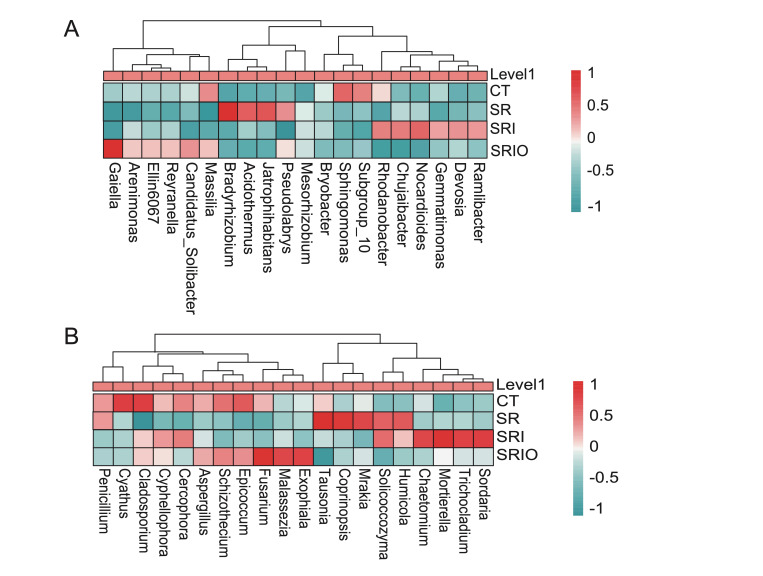
Clustering analysis of microbial communities under four organic amendment strategies. Heatmaps showed the relative abundance of the top 20 microbial taxa at the genus level in (**A**) soil bacteria and (**B**) soil fungi. CT: conventional tillage; SR: straw returning; SRI: straw returning + inorganic fertilizer; SRIO: straw returning + inorganic fertilizer + organic fertilizer.

The results of cluster analysis showed that SR significantly increased the relative abundance of *Bradyrhizobium* by 137.7%, while SRI led to a 140.3% increase in *Nocardioides*. Meanwhile, the *Gaiella* were significantly enriched by 103.9% with the addition of organic fertilizer (SRIO) (Figure 2A). Fungi such as *Tausonia* and *Coprinopsis* were significantly enriched in the SR treatment, while the relative abundance of *Fusarium* was increased by 142.5% in the SRIO treatment (Figure 2B). These results indicated that straw returning affected bacterial and fungal community composition during the maize seedling stage.

### 2.3. The Compositional Differences in the Bacterial and Fungal Community

**Figure 3:**
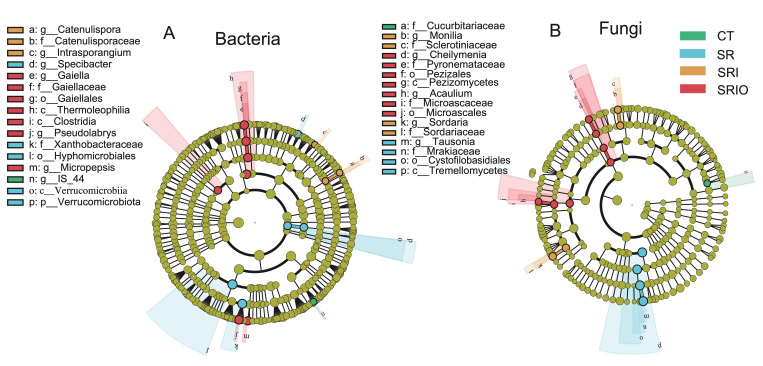
Differential soil microbial taxa under four treatments. Cladograms showed the distribution of bacterial (**A**) and fungal (**B**) taxa with LDA scores > 2 from LEfSe analysis. Multiple testing corrections were applied using the Bonferroni method and FDR adjustment (q < 0.05). Overall differences at the genus level were assessed using the Kruskal–Wallis rank sum test (*p* < 0.05). CT: conventional tillage; SR: straw returning; SRI: straw returning + inorganic fertilizer; SRIO: straw returning + inorganic fertilizer + organic fertilizer.

Linear discriminant analysis effect size (LEfSe) was performed to identify the differential responses of bacterial and fungal communities across four treatments. Significant enrichment of o_Hyphomicrobiales (LDA = 4.30), f_Micrococcaceae (LDA = 3.78), and p_Verrucomicrobiota (LDA = 3.60) was observed in SR treatments. Inorganic fertilizer addition led to the significant enrichment of f_Catenulisporaceae (LDA = 3.40), g_*Catenulispora* (LDA = 3.40), and g_*Intrasporangium* (LDA = 3.75) (SRI treatment). Furthermore, o_Gaiellales (LDA = 4.30), c_Thermoleophilia (LDA = 4.27), and g_*Pseudolabrys* (LDA = 4.06) were identified as significantly enriched taxa in the SRIO treatment (Figure 3A).

f_Mrakiaceae was significantly enriched in the SR treatment (LDA = 5.22). Meanwhile, the inorganic fertilizer application exhibited a significant increase in the relative abundance of g_*Monilia* (LDA = 4.13) and f_Sordariaceae (LDA = 4.28) (SRI treatment). o_Microascales (LDA = 4.45) and f_Pyronemataceae (LDA = 4.28) were significantly enriched in SRIO treatment (Figure 3B).

### 2.4. Analysis of Bacterial and Fungal Community Diversity

**Figure 4:**
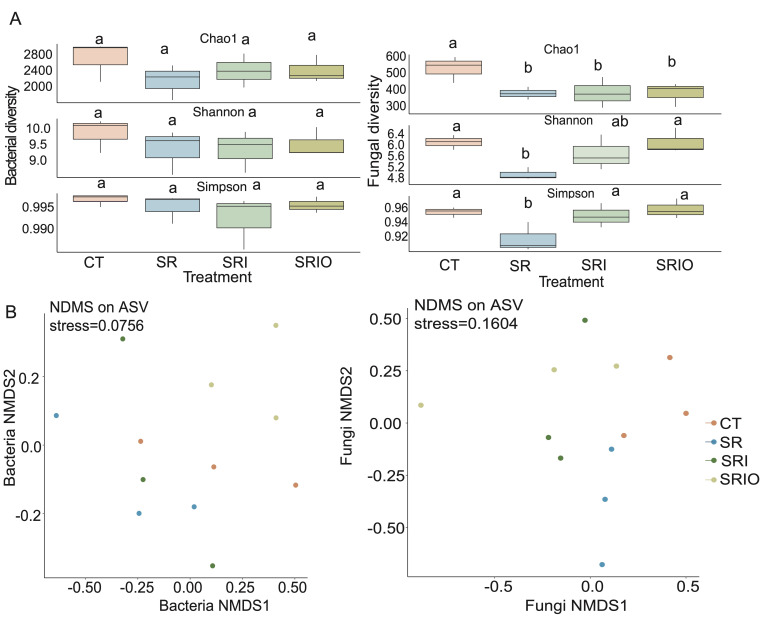
Alpha and beta diversity of soil microbes under four organic amendment strategies. (**A**) Chao1, Shannon, and Simpson indexes (means ± SE, n = 3). Different letters indicate significant differences among treatments (one-way ANOVA, Duncan’s test, *p* < 0.05). (**B**) NMDS ordination of bacterial and fungal communities based on Bray–Curtis distance. CT: conventional tillage; SR: straw returning; SRI: straw returning + inorganic fertilizer; SRIO: straw returning + inorganic fertilizer + organic fertilizer.

The results revealed that straw-amended treatments (SR, SRI and SRIO) had no significant impact on bacterial alpha diversity (Figure 4A). Compared with CT treatments, SR treatment decreased the fungal Chao index by 28.1% (F_3,8_ = 3.1, *p* = 0.089), while the fungal Shannon and Simpson indexes were significantly reduced by 18.8% (F_3,8_ = 4.9, *p* < 0.05) and 4.2% (F_3,8_ = 4.5, *p* < 0.05) (Figure 4A).

The results of non-metric multidimensional scaling (NMDS) based on Bray–Curtis distances revealed that bacterial and fungal communities varied strongly by the treatment of straw returning (Figure 4B). Additionally, significant differences were observed in soil fungal communities among CT, SR, and SRIO treatments (Figure 4B). These findings confirmed that straw-amended treatments had a significant impact on beta diversity.

### 2.5. Co-Occurrence Network Analysis of Soil Bacteria and Fungi

**Figure 5:**
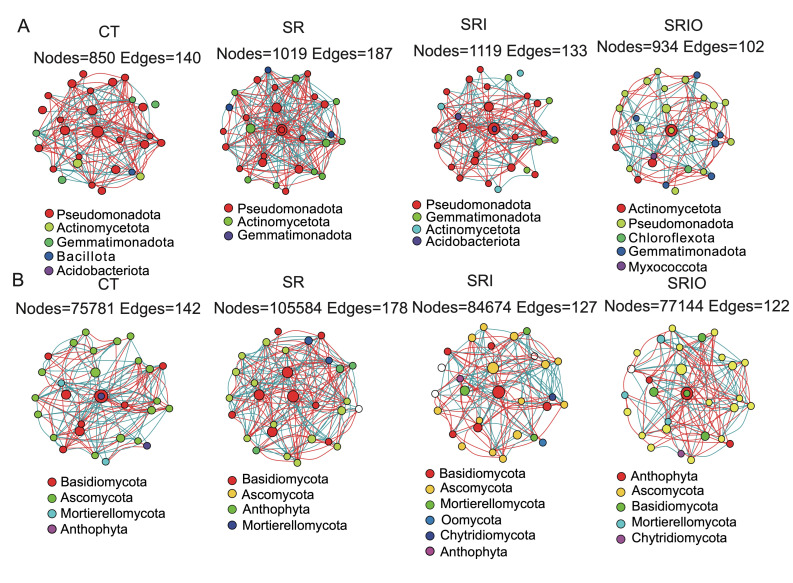
Co-occurrence networks of soil microbial communities under organic amendment strategies. Red edges indicate positive correlations and blue edges indicate negative correlations. Nodes were colored by phylum-level taxonomic assignment. (**A**) Bacterial co-occurrence networks. (**B**) Fungal co-occurrence networks. CT: conventional tillage; SR: straw returning; SRI: straw returning + inorganic fertilizer; SRIO: straw returning + inorganic fertilizer + organic fertilizer.

In this study, co-occurrence analyses were constructed to discuss the microbial coexistence patterns in response to organic amendment strategies (Figure 5). The results showed that under CT treatment, the bacterial co-occurrence network had 850 nodes and 140 edges, both lower than those under straw-amended treatments (Figure 5A). This was accompanied by increased network connectivity and complexity, suggesting that straw returning promoted the interactions of soil bacterial communities (Figure 5A).

For the fungal network, the CT treatment exhibited 75,781 nodes and 142 edges, which was lower than that of straw-amended treatments. Furthermore, a more complex and distinct fungal community structure was observed under SR treatment, while this pattern was diminished following the addition of inorganic fertilizer (SRI) and organic fertilizers (SRIO) (Figure 5B).

### 2.6. Prediction of Soil Bacterial and Fungal Community Functions

**Figure 6:**
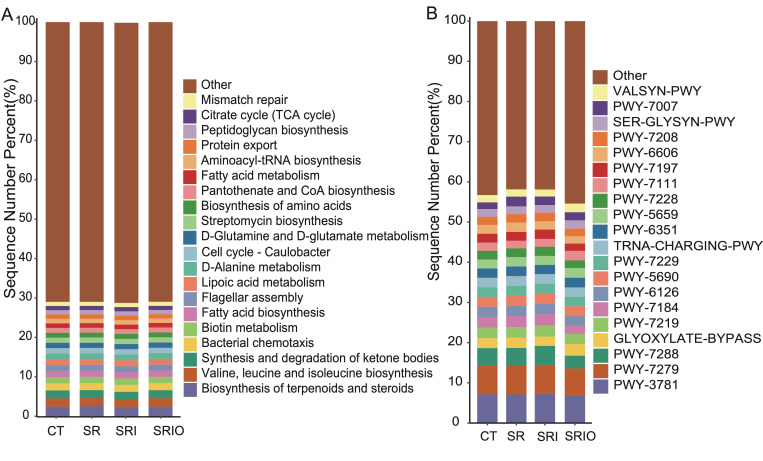
Bar charts showing predicted functions of microbial communities under four organic amendment strategies. (**A**) Predicted functional profile of the bacterial community. (**B**) Predicted functional profile of the fungal community. The metabolic functions of bacteria and fungi were predicted based on ASVs using the PICRUSt2 database with min_identity_to_reference of 0.8. The top 15 KEGG pathways by relative abundance were shown. CT: conventional tillage; SR: straw returning; SRI: straw returning + inorganic fertilizer; SRIO: straw returning + inorganic fertilizer + organic fertilizer.

Functional predictions were further conducted for soil bacteria and fungi, and KEGG metabolic pathways with significant differences were analyzed. The results of bacterial functional prediction using PICRUSt2 showed the SR increased the relative abundance of the terpenoid and steroid biosynthesis pathway by 5.2% (Figure 6A). Moreover, the relative abundance of fungal metabolic pathway PWY-7007 (methyl ketone biosynthesis) was significantly increased by 21.4%, 31.3%, and 16.2% in straw-amended treatments of SR, SRI and SRIO, respectively. Additionally, the metabolic pathway PWY-6606 (guanosine nucleotides degradation II) was significantly reduced by 24.0% in SRIO treatment (Figure 6B).

### 2.7. Relationships Between Microbial Structure and Soil Properties

**Figure 7:**
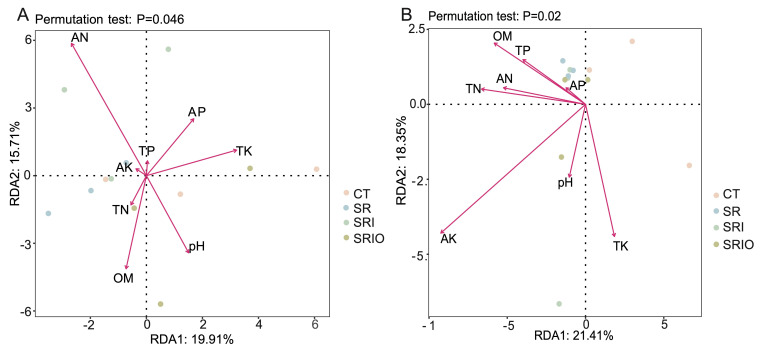
Redundancy analysis (RDA) of soil bacterial community (**A**) and fungal community (**B**) (genus level) and soil chemical properties under four organic amendment strategies. OM: organic matter; TN: total nitrogen; AP: available phosphorus; TP: total phosphorus; AN: alkali nitrogen; AK: available potassium; TK: total potassium. CT: conventional tillage; SR: straw returning; SRI: straw returning + inorganic fertilizer; SRIO: straw returning + inorganic fertilizer + organic fertilizer.

The relationships between microbial communities and soil properties were assessed by redundancy analysis (RDA) analysis. The main drivers responsible for bacterial communities in the SR and SRIO treatments were organic matter (R^2^ = 0.28, *p* = 0.23) and alkali nitrogen (R^2^ = 0.43, *p* = 0.10), and the two axes explain 19.91% and 15.71% of the total variation, respectively (Figure 7A). Moreover, the fungal community composition under SR and SRIO treatments was positively correlated with total nitrogen (R^2^ = 0.33, *p* = 0.17) and available potassium (R^2^ = 0.50, *p* < 0.05), and the two axes explain 21.41% and 18.35% of the total variation, respectively (Figure 7B).

## 3. Discussion

This five-year field study evaluated the effects of organic amendment strategies (SR, SRI, and SRIO) on soil properties and microbial communities in black soil. Results showed that organic materials significantly affected bacterial and fungal community composition, enriching nutrient-cycling taxa including *Bradyrhizobium*, *Nocardioides*, *Gaiella*, *Tausonia*, *Coprinopsis*, and *Fusarium*. While bacterial alpha diversity showed no significant differences among treatments, beta diversity revealed clear separation between straw-amended treatments and CT. The bacterial and fungal communities maintained higher network density in straw returning treatments. Functional prediction indicated that bacterial terpenoid biosynthesis was enriched in SR treatments. This study provides insights for improving the stability of black soil microbial communities through amendments of organic materials.

### 3.1. Organic Amendments Enrich Specific Microbial Taxa Involved in Nutrient Cycling

Our results showed that the addition of maize straw and fertilizer significantly enriched specific bacterial taxa. Previous studies showed that these key taxa were associated with organic matter decomposition and nutrient transformation [19,20]. For instance, *Bradyrhizobium*, a nitrogen-fixing microorganism, was significantly enriched in the SR treatment, suggesting enhanced biological nitrogen fixation [21]. Similarly, *Gaiella*, which was highly correlated with the mineralization of crop straw, was enriched in the SRI and SRIO treatments, respectively. This bacterium can utilize diverse carbon sources to survive in resource-poor environments [22]. Furthermore, studies have shown that *Nocardioides* can effectively degrade the cellulose and lignocellulose present in crop straw [22]. This indicated that organic materials shaped microbial community composition through the selective pressures of their nutrient requirements.

Notably, the biocontrol-associated ASVs, *Tausonia* and *Coprinopsis*, were recruited by maize in SR-related treatments. These beneficial fungi had the potential to suppress pathogens under stress [23]. More importantly, the increase in *Fusarium* abundance under the SRIO treatment raised concern due to its potential pathogenicity. Studies have indicated that the combined application of straw return and organic fertilizer may increase outbreak risks by facilitating the emergence of pathogenic strains [24]. Therefore, the decomposition agent can be applied to accelerate the decomposition of straw, and hot composting can be utilized to eliminate pathogenic fungi.

### 3.2. The Responses of Bacterial and Fungal Diversity to Organic Amendments

Interestingly, bacterial alpha diversity showed no significant differences among treatments, while fungal alpha diversity significantly decreased under SR-related treatments. A similar result was found where straw addition decreased the OTUs, richness, and diversity of bacteria and fungi [25], while a contrasting opinion suggested that straw returning increased bacterial alpha diversity by 3.6% [14]. The concurrent enrichment of biocontrol-associated fungal taxa was a potential driver of the decline in fungal diversity. Notably, these taxa were predominantly K-selected species such as *Trichoderma* and *Tausonia*, which were enhanced in straw-related treatments and dominated the later phase of residue decomposition [26]. Competitive dominance within fungal communities might lead to reduced diversity, thereby shaping community composition.

We found that straw-amended treatments had a significant impact on bacterial and fungal beta diversity. Changes in soil structure and properties drove the distribution of microbial beta diversity. In our study, the increase in soil organic matter content provided sufficient nutrients for microorganism, leading to the rapid proliferation of soil bacteria and fungi [27]. Notably, fungal abundance was more responsive than bacterial abundance to straw returning. Fungi cell walls contained more chemically stable compounds, and fungi played essential roles in low-quality residue decomposition and transformation [28]. Thus, a previous study showed that soil fungi, rather than bacteria, were primarily responsible for carbon stabilization through residue [28]. These results suggested that the application of organic amendments could contribute to the stability of the soil ecosystem.

### 3.3. Organic Amendments Enhance Microbial Network Complexity and Stability

The co-occurrence analysis revealed that SR-related treatments substantially increased both connections (edges) and taxonomic units (nodes) of the microbial network (Figure 5). This enhanced network complexity suggested that organic materials supported stronger cooperative relationship between microorganisms [29]. Studies have demonstrated that straw returning created favorable habitats for microorganisms, thereby mitigating the adverse effects of external disturbances to community stability [30,31]. The diverse ecological niches provided by additional nutrients allowed for the coexistence of microbial taxa with complementary metabolic capabilities [16]. This research showed that straw returning can increase the stability and resilience of microbial communities, thereby further promoting black soil health.

We observed different network patterns between bacterial and fungal communities in response to organic materials. For bacteria, all straw-amended treatments exhibited similarly complex networks, suggesting that straw carbon was the primary driver of bacterial network complexity [32]. In contrast, fungal network complexity was highest under SR treatment and was reduced by the addition of inorganic (SRI) and organic fertilizers (SRIO). This may reflect the greater sensitivity of fungi to nutrient competition than bacteria [33]. Fungi played important roles in decomposing complex organic matter such as cellulose and hemicellulose in straw [34]. The addition of readily available nutrients may disturb a competitive equilibrium by favoring specific fungal functional groups [27], thereby simplifying the fungal co-occurrence network. This finding highlighted the distinct responses of bacterial and fungal communities to resource availability.

### 3.4. Soil Chemical Properties Drive Shifts in Microbial Community Structure

Redundancy analysis revealed that soil organic matter, alkali nitrogen, and available potassium were the primary environmental factors shaping both bacterial and fungal community structures (Figure 7). Previous studies demonstrated that organic carbon and nutrient availability were dominant drivers of microbial community assembly in agricultural soils [35]. In our study, straw returning directly increased soil organic carbon contents, thereby providing sustained carbon sources for heterotrophic microorganisms (Figure 1) [36]. Straw inputs typically had high C/N ratios, which can reshape the composition and diversity of soil microorganisms [37]. For instance, the enrichment of *Bradyrhizobium* under SR treatment may represent microbial strategies to alleviate nitrogen limitations imposed by straw decomposition (Figure 2A). In our study, straw returning resulted in elevated levels of soil organic matter, alkali nitrogen, and available potassium, which positively influenced the abundance of nitrogen-cycling microorganisms [38,39]. This explained the distinct community separation observed between straw-amended treatments and CT in the NMDS ordination (Figure 4B).

## 4. Materials and Methods

### 4.1. Experimental Design

A five-year field experiment was conducted in Northeast China (124.4° E, 43.4° N) since 2020. This area has an annual precipitation level of 600 mm and annual average temperature of 6.0 °C. The soil type is black soil, which has a black surface horizon and is enriched with organic matter. Different organic amendment strategies are described as follows: (1) conventional tillage (CT); (2) straw returning (SR); (3) straw returning + inorganic fertilizer (SRI); and (4) straw returning + inorganic fertilizer + organic fertilizer (SRIO). Conventional tillage comprised ridge tillage without straw, whereas straw returning involved no tillage with straw mulch. Throughout the experimental period, maize was cultivated as a single annual crop under a continuous monocropping system. Fumin-1433 maize was selected for the experiments, and the average maize straw yield in the area was approximately 6 t·ha^−1^. The organic fertilizer used in this study was an aerobic compost prepared from cow manure (provided by farmers, N:P_2_O_5_:K_2_O = 0.59:0.28:0.14), while the inorganic fertilizer was a compound fertilizer (10% N, 25% P_2_O_5_, 25% K_2_O). In this study, the inorganic fertilizer was applied at application rates of 300 kg·ha^−1^, while the organic fertilizer was applied at the application rate of 1000 kg·ha^−1^. The inorganic and organic fertilizers were spread on the surface soil before sowing and were then harrowed into the topsoil (0–30 cm); no topdressing measures were applied in this experiment. Maize straws were chopped into 5–10 cm fragments after harvest and were applied to the soil surface under no-tillage conditions for five consecutive years (full return of straw to the field).

**Figure 8:**
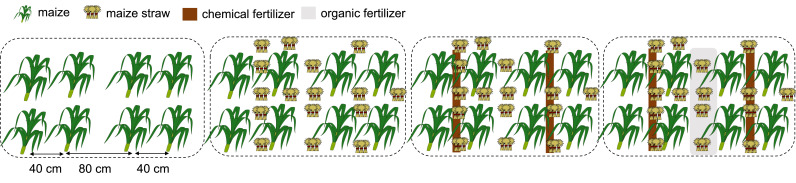
Schematic overview of the experimental design. A five-year field experiment was conducted with four treatments: (1) conventional tillage (CT); (2) straw returning (SR); (3) straw returning + inorganic fertilizer (SRI); and (4) straw returning + inorganic fertilizer + organic fertilizer (SRIO). A randomized complete block design was employed with three replications per treatment (n = 3). Each plot covered an area of 750 m^2^ (50 m × 15 m). Maize was cultivated using an alternating wide-narrow row planting pattern, and all straw was returned to the field under no-till conditions.

This study employed a wide-narrow row planting pattern, with a wide row spacing of 80 cm and a narrow row spacing of 40 cm between adjacent maize rows [40]. The experiments were laid out using a randomized complete block design with three replicates. The maize was planted in six rows per experimental block, with each block covering 750 m^2^ (50 m × 15 m) within the cropping system. The planting density was 75,000 plants·ha^−1^. A 2 m wide guard row was maintained between the blocks. The inorganic fertilizer was applied to the narrow rows where maize was planted and organic fertilizer to the wide rows (Figure 8). Under different treatments, irrigation, nutrient management, and pest control followed farming practices. The initial nutrient concentrations constituting 0–20 cm of soil were as follows: pH, 5.12; soil organic matter, 9.6 g·kg^−1^; total nitrogen, 1.2 g·kg^−1^; available phosphorus, 8.4 mg·kg^−1^; available phosphorus, 101.0 mg·kg^−1^. We collected soil samples from each plot to assess both the chemical properties and microbial communities of maize growth at 45 days.

### 4.2. Sample Collection

Soil samples were collected from the 0–20 cm layer using a five-point sampling method and thoroughly mixed for subsequent analyses, and the five sampling points were spaced 10 m apart. Each mixed sample was first passed through a 2 mm sieve to remove roots and large debris. The sieved soil was then divided into two portions: one was stored at room temperature for determination of soil chemical properties, and the other was stored at −80 °C for subsequent microbial community analysis.

#### 4.2.1. Analysis of Soil Chemical Properties

Measurements of the soil chemical characteristics were conducted as follows: The soil pH value was measured using a pH meter (Sedoris PB-10) at a soil-to-water ratio of 1:2.5; the organic matter was determined by the hydrated heat potassium dichromate oxidation-colorimetry method (the conversion factor was 1.724; the oxidation correction coefficient was 1.1); the total nitrogen was tested by Kjeldahl method; the total and available phosphorus were analyzed by alkali fusion-Mo-Sb anti spectrophotometric method; total phosphorus was determined using HNO_3_-HClO_4_-HF mixture as the solvent and a melting temperature of 200 °C; the available potassium and total potassium was determined by flame spectrophotometry; the specific solvents was HNO_3_-HClO_4_-HF mixture and the melting temperatures was 200 °C for the determination of total potassium; and the soil alkali nitrogen was measured using alkaline hydrolysis diffusion separation acid–base titration method.

#### 4.2.2. DNA Extraction, Amplification, and High-Throughput Sequencing

DNA was extracted using a magnetic bead-based soil DNA extraction kit (Catalog number: DP712-01; Batch number: DP180427) following the manufacturer’s instructions (Tiangen Biotech (Beijing) Co., Ltd., Beijing, China). The concentration of extracted DNA was determined using a NanoDrop spectrophotometer (Thermo Fisher Scientific, Waltham, MA, USA), and the DNA was subsequently stored at −20 °C. Bacteria were identified using 16S V4-V7 region primers (GTGCCAGCMGCCG CGGTAA, GGACTACHVGGGTWTCTAAT), and fungi were identified using ITS1 region primers (GGAAGTAAAAGTCGTAACAAGG, GCTGCGTTCTTCATCGATGC) [41]. The PCR reactions had a total volume of 30 μL, consisting of 15 μL Phusion Master Mix (2×) (Takara Bio, Tokyo, Japan), 1 μL each of forward and reverse primers (1 μM·μL^−1^), 10 μL of DNA template (1 ng·μL^−1^), and 2 μL of ddH_2_O. Thermal cycling consisted of initial denaturation at 98 °C for 1 min, followed by 30 cycles of 98 °C for 10 s, 50 °C for 30 s, and 72 °C for 30 s, and a final elongation at 72 °C for 5 min. PCR products were pooled in equal amounts based on concentration, thoroughly mixed, and then purified using 1× TAE 2% agarose gel electrophoresis. The target fragments were recovered using universal DNA purification and recovery kit (Tiangen Biotech (Beijing) Co., Ltd., Beijing, China). Library construction was performed using the NEB Next^®^ Ultra DNA Library Prep Kit (Tiangen Biotech (Beijing) Co., Ltd., Beijing, China). We used q-PCR to quantify and assess the quality of the constructed library. Libraries that passed QC were then sequenced on the Illumina platform.

#### 4.2.3. Sequence Processing

Paired-end sequencing reads were processed using QIIME 2 (see qiime.org) [30]. Raw sequences were demultiplexed through quality filtering and denoising using the QIIME2 dada2 plugin. The amplicon sequence variants (ASVs) were then aligned against the GREENGENES database to generate the taxonomy table. All chloroplast, mitochondrial, and archaeal sequences were subsequently removed [42]. Subsequently, all samples were rarefied to the minimum sequencing depth, ultimately retaining 589,236 bacterial ASVs and 1,000,043 fungal ASVs for downstream analysis.

### 4.3. Statistical Analysis

Diversity metrics were calculated using the core-diversity plugin within QIIME2. The Chao1, Simpson, and Shannon indexes were calculated to estimate the alpha diversity within an individual sample. Beta diversity was visualized using NMDS based on Bray–Curtis distance. LEfSe was used to identify significantly different ASVs (*p* < 0.05, LDA > 2). The potential KEGG Ortholog (KO) functional profiles of microbial communities were predicted with the PICRUSt2 database [43]. The effect of organic amendment strategies on soil chemical properties and microbial alpha diversity was assessed with one-way analysis of variance (ANOVA), and differences among treatments were further assessed with Duncan’s multiple range test. Data were tested for homogeneity of variance prior to analysis and log-transformed if necessary.

To investigate co-occurrence network patterns and identify keystone taxa in bacterial and fungal communities under four treatments, separate networks were constructed for each treatment using ASVs with relative abundance > 0.01%. Spearman’s correlations were calculated using the cor.test function in R 4.5.0, with *p*-values adjusted by the FDR method. The R package igraph was used to construct networks based on the correlation matrix, retaining only robust (|r| > 0.4) and significant (adjusted *p* < 0.05) correlations. Network visualization was performed with Gephi 0.9.2, and topological properties were calculated using igraph 0.9.8. In addition, the relationship between soil properties and microbial community composition was analyzed using a redundancy analysis (RDA).

## 5. Conclusions

Organic amendment strategies, especially straw returning combined with fertilizers, significantly influenced soil microbial community composition, network complexity, and metabolic functions in black soil. Soil organic matter, alkali nitrogen, and available potassium played a predominant role in the assembly of bacterial and fungal communities under straw-amended treatments. This study showed that organic materials can improve soil properties and enhance the stability of the soil ecosystem. However, different ratios of maize straw and organic fertilizer need to be explored to reduce the addition of chemical fertilizers. In conclusion, our research provided sustainable management practices to restore degraded black soil. Future research should investigate the effects of organic materials on functional genes involved in nitrogen and carbon mineralization, which will benefit black soil conservation.

## Data Availability

The original contributions presented in this study are included in the article material. Further inquiries can be directed to the corresponding author.

## References

[1] Xiao Y., Luo W., Yang K., Fu J., Wang P. (2025). Plow tillage with buried straw increases maize yield by regulating soil properties, root growth, photosynthetic capacity, and bacterial community assembly in semi-arid black soil farmlands. Eur. J. Agron..

[2] Ding J., Li Z., Wu J., Ma D., Chen Q., Li J. (2025). Effects of short-term straw return and manure fertilization on soil microorganisms and soybean yield in parent material of degraded black soil in Northeast China. Microorganisms.

[3] Chu J., Wang L., Jia R., Zhou J., Zang H., Wang J., Yang Y., Jiang Y., Wang Y., Peixoto L. (2024). Straw returning with no-tillage alleviates microbial metabolic carbon limitation and improves soil multifunctionality in the northeast plain. Land Degrad. Dev..

[4] Kang Z., Li N., Han X., Wang C., Yue J., Yu H. (2025). Synergistically enhanced black soil conservation by *Paenarthrobacter* sp. *KN*0901 under straw amendment: Dual promotion of atrazine degradation and nutrient retention. Environ. Res..

[5] Chen L., Liu Q., Du H., Cui J., Chen Y. (2024). Organic materials return suppressed soil N_2_O emissions by changing the composition instead of abundance of denitrifying microbial community. Appl. Soil Ecol..

[6] Wang X., Li X., Wang Z., Long A., Ji X., Gong X., Jiang Y., Qi H. (2025). Straw return increased maize phosphorus uptake and grain yield by alleviating rhizosphere soil microbial metabolism limitation: Insights from ecoenzymatic stoichiometry. Plant Soil.

[7] Wang Y., Zhang Y., Liu Y., Wang L., Dong Y. (2024). Effects of different tillage methods on soil properties and maize seedling growth in alternating wide and narrow rows rotation mode in the Songliao Plain of China. Geoderma.

[8] Xing S., Zhang G., Chen S., Zhang N., Wang C. (2024). Response of soil erosion resistance to straw incorporation amount in the black soil region of Northeast China. J. Environ. Manag..

[9] Dămătîrcă C., Moretti B., Bertora C., Ferrarini A., Lerda C., Mania I., Celi L., Gorra R., Zavattaro L. (2023). Residue incorporation and organic fertilisation improve carbon and nitrogen turnover and stabilisation in maize monocropping. Agric. Ecosyst. Environ..

[10] Liu M., Zhang Z., He P., Zhang Y., Li L.-J. (2025). Changes in soil microbial community and carbon use efficiency in freeze-thaw period restored after growth season under warming and straw return. Appl. Soil Ecol..

[11] Bender S.F., Wagg C., van der Heijden M.G.A. (2016). An underground revolution: Biodiversity and soil ecological engineering for agricultural sustainability. Trends Ecol. Evol..

[12] Cong P., Wang J., Li Y., Liu N., Dong J., Pang H., Zhang L., Gao Z. (2020). Changes in soil organic carbon and microbial community under varying straw incorporation strategies. Soil Tillage Res..

[13] Xu H., Sun J., Zhao Z., Gao Y., Tian L., Wei X. (2025). Long-term straw return promotes soil phosphorus cycling by enhancing soil microbial functional genes responsible for phosphorus mobilization in the rice rhizosphere. Agric. Ecosyst. Environ..

[14] Wu G., Ling J., Zhao D.-Q., Liu Z.-X., Xu Y.-P., Kuzyakov Y., Marsden K., Wen Y., Zhou S.-L. (2023). Straw return counteracts the negative effects of warming on microbial community and soil multifunctionality. Agric. Ecosyst. Environ..

[15] Li W., Liu X., Xia Q., Gao Z., Zheng W., Zhai B., Yang Z. (2024). Untargeted metabolomics to study changes in soil microbial community in response to tillage practices. Appl. Soil Ecol..

[16] Hu Y., Li Y., Liu K., Shi C., Wang W., Yang Z., Xu K., Li S., Wang Y., Jin L. (2025). Improving the stability of black soil microbial communities through long-term application of biochar to optimize the characteristics of DOM components. Biochar.

[17] Zhang Y., Osborne B., Dang S., Zou J. (2025). The effects of straw return and tillage depth on soil respiration and soil organic carbon: Implications for improving the sustainability of agro-ecosystems in China. Eur. J. Agron..

[18] Wang W., Ding S., Guo T., Xu X., He P., Huang S. (2026). Organic amendment strategies differentially regulate microbial carbon use efficiency: A long-term field study integrating microorganism and enzymatic stoichiometry. Agric. Ecosyst. Environ..

[19] Xu J., Song F., Wang Z., Qi Z., Liu M., Guan S., Sun J., Li S., Zhao J. (2024). Effects of different straw return methods on the soil structure, organic carbon content and maize yield of black soil farmland. Agronomy.

[20] Chen H., Duan M., Wang Q., Zhou B., Yan R., Chen X., Deng M. (2026). Organic–mineral fertilization modulates microbial communities and nutrient-cycling genes in saline–alkali soil. Front. Microbiol..

[21] Xu L., Zhou Y., Miao C., Zhang J., Ding Y., Liu Z., Li W., Jiang Y., Li G. (2025). The coupling effects of long-term straw return and plant selection facilitate rhizosphere nitrogen supply by promoting recruitment of core genera. Appl. Soil Ecol..

[22] Chen X., Xu Y., Sun R., Ye X., Ma C., Mao J., Zhang C., Gao H., Zhang W. (2023). Soil microbial communities under wheat and maize straw incorporation are closely associated with soil organic carbon fractions and chemical structure. Appl. Soil Ecol..

[23] Fan Y., Liu J., Liu Z., Gu H., Hu X., Yu Z., Li Y., Jin J., Liu X., Wang G. (2026). Soil amendments alleviate continuous cropping obstacles in soybean by enhancing microbial resistance. Field Crops Res..

[24] Hao M., Hu H., Liu Z., Dong Q., Sun K., Feng Y., Li G., Ning T. (2019). Shifts in microbial community and carbon sequestration in farmland soil under long-term conservation tillage and straw returning. Appl. Soil Ecol..

[25] Zhao S., Qiu S., Xu X., Ciampitti I.A., Zhang S., He P. (2019). Change in straw decomposition rate and soil microbial community composition after straw addition in different long-term fertilization soils. Appl. Soil Ecol..

[26] Zhou T., Zang Y., Li Z., Zhang Y., Zhu K., Zhang W., Zhang H., Liu L., Wang Z., Gu J. (2025). Controlled-release nitrogen fertilizer and long-term straw return synergistically improve wheat yield and reduced the nitrogen losses by regulating soil microbial communities and soil organic nitrogen components. Field Crops Res..

[27] Li W., Qu W., Zong R., Li J., Ho S.H., Wang Z. (2024). Tillage with straw returning promotes soil functional microbiomes and nitrogen transformation to increase cotton yield. Land Degrad. Dev..

[28] Zhu X., Xie H., Masters M.D., Rui Y., Luo Y., He H., Zhang X., Liang C. (2023). Microorganisms, their residues, and soil carbon storage under a continuous maize cropping system with eight years of variable residue retention. Appl. Soil Ecol..

[29] Wei Z., Han X., Wang Y., Zhang L., Gong P., Shi Y. (2025). Effects of biochar, dual inhibitor, and straw return on maize yield, soil physicochemical properties, and microbial system under fertilization conditions. Front. Microbiol..

[30] Sun N., Zhao X., Liu F., Song G., Zhang M., Song F. (2025). Land use change has profoundly altered the process of bacterial community assembly in the northeastern black soil zone. Front. Microbiol..

[31] Huang S., Gao X., Zeng L., Zhang M., Zhang L., Wang S., Zhao Y., Zhou W., Ai C. (2024). Microbial role in enhancing transfer of straw-derived nitrogen to wheat under nitrogen fertilization. Soil Tillage Res..

[32] Ma C., He Z., Xiang J., Ding K., Zhang Z., Ye C., Wang J., Kalkhajeh Y.K. (2025). A meta-analysis to explore the impact of straw decomposing microorganism inoculant-amended straw on soil organic carbon stocks. J. Integr. Agr..

[33] Luo Y., Zhang L., Wang Y., Huang W., Lu Y., Song S., Zhu J., Zhou H., Su D., Zheng D. (2026). Rhizosphere soil fertility and microbial community characteristics of *Arundo donax* cv. Lvzhou No.1 in coastal saline-alkali soils. Front. Plant Sci..

[34] Qiao Y., Xu D., Peng J., Lu H., Tan Y., Guo D. (2024). Influence of decomposed stubble return on the soil microbial community under perennial crop rotation. J. Soil Sci. Plant Nutr..

[35] Yan W., Xia M., Liu J., Han Z., Li Z., Rensing C., Alwathnani H.A., Chen B., Wu W., Wu H. (2026). Straw return improves soil multifunctionality by altering functional microbial diversity and abundance. Agric. Ecosyst. Environ..

[36] Pei H., Miao Y., Liang A., Liu Q., Hou R. (2025). Improving cropland soil water management to promote soil organic carbon increase through organic material returning in cold black soil areas. Agric. Ecosyst. Environ..

[37] Berhane M., Xu M., Liang Z., Shi J., Wei G., Tian X. (2020). Effects of long-term straw return on soil organic carbon storage and sequestration rate in North China upland crops: A meta-analysis. Glob. Change Biol..

[38] Zeng X., Wang Q., Song Q., Liang Q., Sun Y., Song F. (2025). Effects of different nitrogen fertilizer application rates on soil microbial structure in paddy soil when combined with rice straw return. Microorganisms.

[39] Oliverio A.M., Bissett A., McGuire K., Saltonstall K., Turner B.L., Fierer N. (2020). The role of phosphorus limitation in shaping soil bacterial communities and their metabolic capabilities. mBio.

[40] Struik P.C., Feng L., Raza M.A., Chen Y., Khalid M.H.B., Meraj T.A., Ahsan F., Fan Y., Du J., Wu X. (2019). Narrow-wide row planting pattern improves the light environment and seed yields of intercrop species in relay intercropping system. PLoS ONE.

[41] Jiang P., Wang Y., Zhang Y., Fei J., Rong X., Peng J., Yin L., Luo G. (2024). Intercropping enhances maize growth and nutrient uptake by driving the link between rhizosphere metabolites and microbiomes. New Phytol..

[42] Yuan C., Ma Z., Liu S., Nie H., Feng G., Wang S., Luo S. (2025). Effects of strip-tillage on soil microbial community structure and function in black soil. Front. Microbiol..

[43] Wan Y., Ma Z., Lang J., Xu X., Shao C., Chen J., Ge T., Zhang H. (2025). Host genotype-driven assembly of bacterial communities in the rice root microdomains. Soil Ecol. Lett..

